# Cannabidiol enhances cytotoxicity of anti-cancer drugs in human head and neck squamous cell carcinoma

**DOI:** 10.1038/s41598-020-77674-y

**Published:** 2020-11-26

**Authors:** Yoon Young Go, Su Ra Kim, Do Yeon Kim, Sung-Won Chae, Jae-Jun Song

**Affiliations:** 1 Department of Otorhinolaryngology-Head and Neck Surgery, Korea University Guro Hospital, 80 Guro-dong, Guro-gu, Seoul, 08308 South Korea; 2 Institute for Health Care Convergence Center, Korea University Guro Hospital, Seoul, Republic of Korea

**Keywords:** Head and neck cancer, Medical research

## Abstract

Cannabidiol (CBD) has anti-tumorigenic activity. However, the anti-cancer effect of CBD on head and neck squamous cell carcinoma (HNSCC) remains unclear. The cytotoxicity of CBD on HNSCC was analyzed using cell survival and colony-forming assays in vitro. RNA-seq was used for determining the mechanism underlying CBD-induced cell death. Xenograft mouse models were used to determine CBD’s effects in vivo. CBD treatment significantly reduced migration/invasion and viability of HNSCC cells in a dose- and time-dependent manner. HNSCC mouse xenograft models revealed anti-tumor effects of CBD. Furthermore, combinational treatment with CBD enhanced the efficacy of chemotherapy drugs. Apoptosis and autophagy processes were involved in CBD-induced cytotoxicity of HNSCCs. RNA-seq identified decreased expression of genes associated with DNA repair, cell division, and cell proliferation, which were involved in CBD-mediated cytotoxicity toward HNSCCs. We identified CBD as a new potential anti-cancer compound for single or combination therapy of HNSCC.

## Introduction

Head and neck squamous cell carcinoma (HNSCC) is a malignant tumor that occurs in widespread areas of the head and neck region, including the oral and nasal cavities, salivary glands, pharynx, and larynx, with a heterogeneous mixture of upper aerodigestive tract malignancies^1^. HNSCC is considered the sixth most common cancer, accounting for approximately 4% of all cancers worldwide, and the 5-year survival rate in patients with late-stage HNSCC is less than 50%^2^. Currently, chemotherapy with cisplatin, methotrexate, and 5-fluorouracil is applied in patients with HNSCC as single agents or in combination with radiotherapy to reduce the initial tumor area before physical surgery^3^. Chemotherapeutic agents, especially platinum compounds (Cisplatin), taxanes (Paclitaxel), and 5-Fluorouracil are well-known effective drugs for cancer treatment. However, use of chemotherapeutic agents cause undesirable side effects such as severe hair loss, nausea, vomiting, kidney problem, and decreased immunity to infections as well as drug resistance^4,5^. Therefore, mono-targeted therapies based on molecular research to treat HNSCC have been developed and tested in clinical studies, the FDA has approved several single agents for HNSCC including cetuximab and pembrolizumab^6,7^. Although cetuximab was specifically developed to target the epidermal growth factor receptor (EGFR), which is overexpressed in HNSCC, low treatment response and side effects such as acquired resistance often limit its application in chemotherapy for all patients with HNSCC^6^. Therefore, more effective agents are needed for chemotherapy strategies in patients with HNSCC.

Cannabidiol (CBD) is one of the components in the *Cannabis sativa* L. (marijuana) family of plants, and basic research has been elucidating the chemical structure of CBD compounds since the 1960s^8^. Cannabis plants consist of two major components, CBD and tetrahydrocannabinol (THC), which have different bioactive and medicinal effects on the human body. For example, THC has been well known for its psychoactive and addictive properties, whereas CBD attenuates the psychoactivity of THC in the nervous system^8^. There are concerns about CBD’s effects on physical and mental health; yet, preclinical studies suggest there are medical benefits of CBD for treating drug and tobacco addiction in humans^9^. Therefore, CBD is considered a safe pharmacological constituent of the cannabinoid family, which is known to have various medicinal effects, including anti-inflammatory and anti-ischemic effects, plus potential to treat neuropathic pain and childhood seizure disorders^10–12^. Currently, CBD is widely consumed as an extract or oil from cannabis plants in European countries and America^12^.

CBD has potential benefits for treating cancer, including extensive inhibition of tumor growth, angiogenesis, and metastasis in various cancer models^13,14^. The molecular mechanism underlying the anti-cancer effect of CBD is not fully understood, but the majority of studies have shown that it inhibits cancer cell proliferation via apoptosis signaling^15^. In addition, one of the most well-known advantages of CBD regarding its medicinal effects is cancer-associated pain relief. Compared with side effects of chemo-anticancer drugs, CBD can effectively alleviate nausea and vomiting induced by chemotherapy during cancer therapy^16^. Therefore, CBD can have a dual advantage for cancer therapy. Recently, it has been reported that cannabinoids can induce the carcinogenesis of human papillomavirus (HPV)-positive HNSCC^17^. However, it is still unclear the effects of CBD on HPV-negative HNSCC. In this study, we investigated the mechanisms underlying CBD-induced cell death in HPV-negative HNSCC cell lines, and determined the synergistic efficacy of combining chemotherapy agents with CBD. Additionally, we also provide in vivo evidence that CBD reduces head and neck tumor growth as an effective cytotoxic agent against HNSCC.

## Results

### Cytotoxic effect of CBD on HNSCC cells

**Figure 1:**
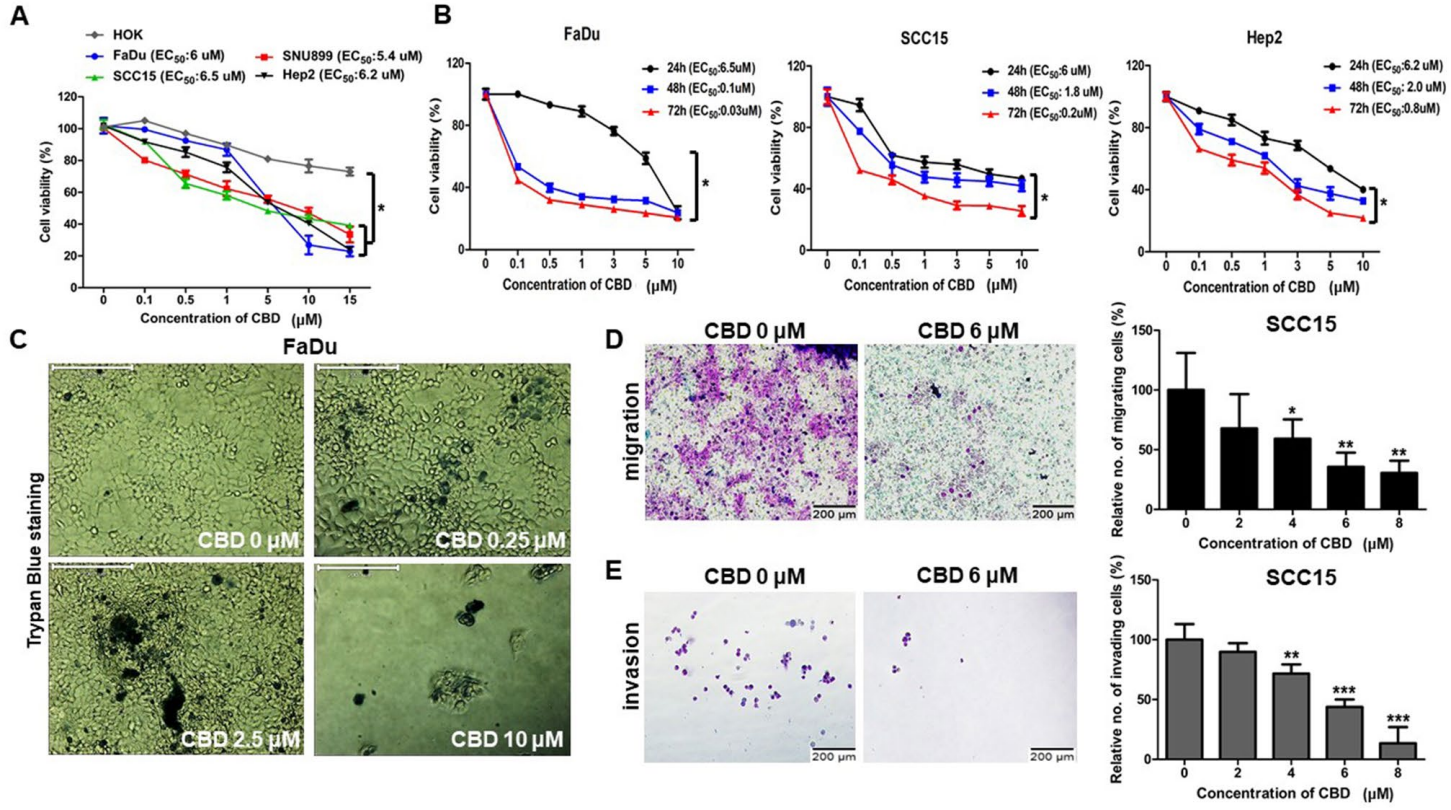
Cannabidiol (CBD) induced cytotoxicity in head and neck squamous cell carcinoma (HNSCC) cells. (**A**) A normal cell line (HOK) and HNSC cells (FaDu, SNU899, SCC15, Hep2) were treated with various concentrations (0, 0.1, 0.5, 1, 5, 10, 15 µM) of CBD for 24 h and then cell viability was determined by CCK8 assay. EC_50_ indicates the efficient concentration for 50% cell death. (**B**) FaDu, SCC15, and Hep2 cells were treated with 0, 0.1, 0.5, 1, 3, 5, and 10 µM of CBD. The cell viability was measured by CCK8 assay at 24, 48, and 72 h. (**C**) FaDu cells were seeded in a 24-well plate and then exposed to CBD in each growth medium for 48 h. Cells stained with 0.4% trypan blue solution were observed by light microscopy. The scale bar represents 200 μm. (**D**,**E**) SCC15 cells were exposed to 0 or 6 µM of CBD for 24 h and then added cells into Matrigel-coated or uncoated transwell upper inserts in serum-free media. After incubation for 24 h, transmigrated cells were stained by Diff Quick solution and then visualized by light microscopy. The migrating and invading cells were counted for each group. Scale bars, 200 μm. Data were represented as mean and standard deviation (SD) (n = 3); *p < 0.05; **p < 0.01; and ***p < 0.001 compared with the corresponding control.

To investigate the cytotoxic effect of CBD on human HNSCC cells, we first measured the cell viability of four HNSCC cell lines using CCK8 assays after treatment with various concentrations of CBD (0–15 µM) for 24 h. A greater decrease in viability was detected in HNSCC cells cultured with CBD (< 6 µM) than in normal human oral keratinocytes (HOK) (Fig. 1A). In addition, viability of HNSCCs was significantly suppressed by CBD in a time-dependent manner upon treatment for 48–72 h, indicating the significant cytotoxicity of CBD as an anti-cancer drug (Fig. 1B). Similar to the CCK8 assay results, trypan blue exclusion assay results also showed induction of HNSCC cell death at day 2 under treatment with different concentrations of CBD, suggesting that CBD has anti-cancer potential in HNSCC cells (Fig. 1C). We then investigated whether CBD has anti-migration and anti-invasion effects on human HNSCC cells (SCC15 cells). For migration and invasion assays, CBD-treated and -untreated SCC15 cells were allowed to transmigrate in transwell insert systems and then the number of migrated/invaded cells was determined. The number of transmigrated SCC15 cells significantly decreased upon CBD treatment in a dose-dependent manner, compared with that for the control SCC15 cells in both migration and invasion assay systems (Fig. 1D,E). These results demonstrated that CBD inhibits the migration and invasion of human HNSCC cells and also has cytotoxic effects.

### Anti-tumor effect of CBD in vivo

**Figure 2:**
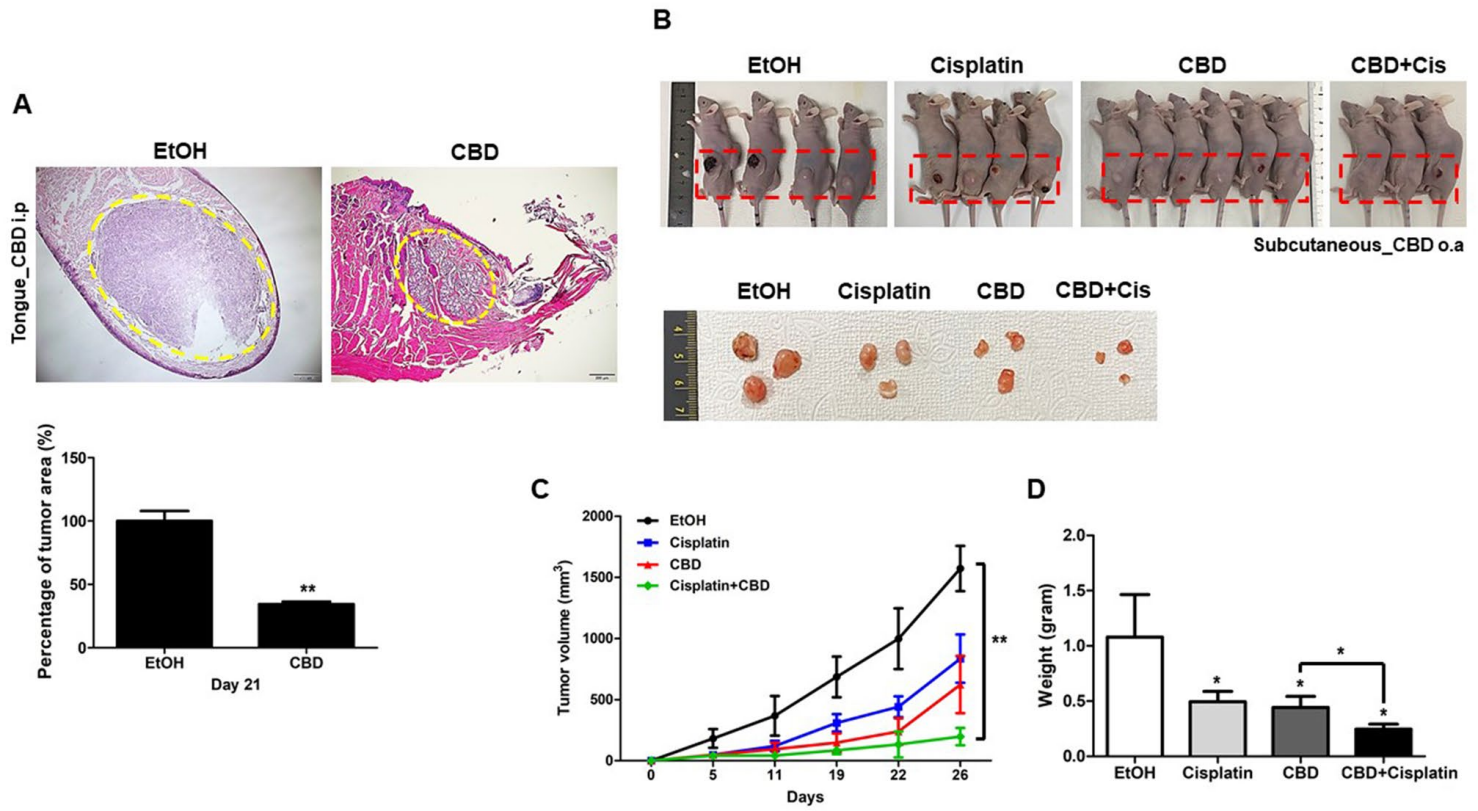
Anti-tumor effect of cannabidiol (CBD) in a head and neck squamous cell carcinoma (HNSCC) xenograft animal model. (**A**) FaDu cells (2 × 10^6^) injected into tongues of nude mice. After 2–3 days, mice were treated with EtOH or CBD (5 mg/kg) or intraperitoneally (i.p). H&E staining of FaDu cell xenograft mice (tongue) treated with CBD or EtOH after 3 weeks were observed by light microscopy and tumor size was measured. The percentage of tumor area was determined by Image J software. Scale bars represent 200 μm. (**B**) FaDu cells (2 × 10^6^) injected subcutaneously to the animal. FaDu cell xenograft mice (Subcutaneous) treated with EtOH, CBD (5 mg/kg), Cisplatin (2.5 mg/kg) or CBD (5 mg/kg)/Cisplatin (2.5 mg/kg) (CBD + Cis) after 4 weeks were sacrificed and the photographs of mice and tumors were taken using a digital camera. EtOH or CBD were orally (o.a) treated and Cisplatin was intraperitoneally (i.p) injected. Tumor volume (**C**) and weight (**D**) were statistically determined. (n = 4 mice/group and 3 images/mouse) Data were represented as mean and SD; *p < 0.05, and **p < 0.01 compared with the corresponding control.

Next, we further validated the anti-tumor capacity of CBD using a xenograft mouse model. Two different FaDu cell xenograft mouse models were generated by injecting 2 × 10^6^ cells subcutaneously or into tongues of BALB/c nude mice. As shown in Fig. 2A, tumor sizes of tongue xenograft tumors were significantly decreased in CBD-treated mice compared with those in untreated control mice. This result indicated that CBD inhibited the growth of HNSCC cells in vivo. Moreover, remarkable inhibitions of tumor growth and weight were observed in mice that received combination therapy with both CBD and Cisplatin (Cis) (Fig. 2B–D), which means that CBD can be used in combination with general anti-cancer drugs such as Cis.

### CBD enhances the effect of anti-cancer drugs in HNSCCs

**Figure 3:**
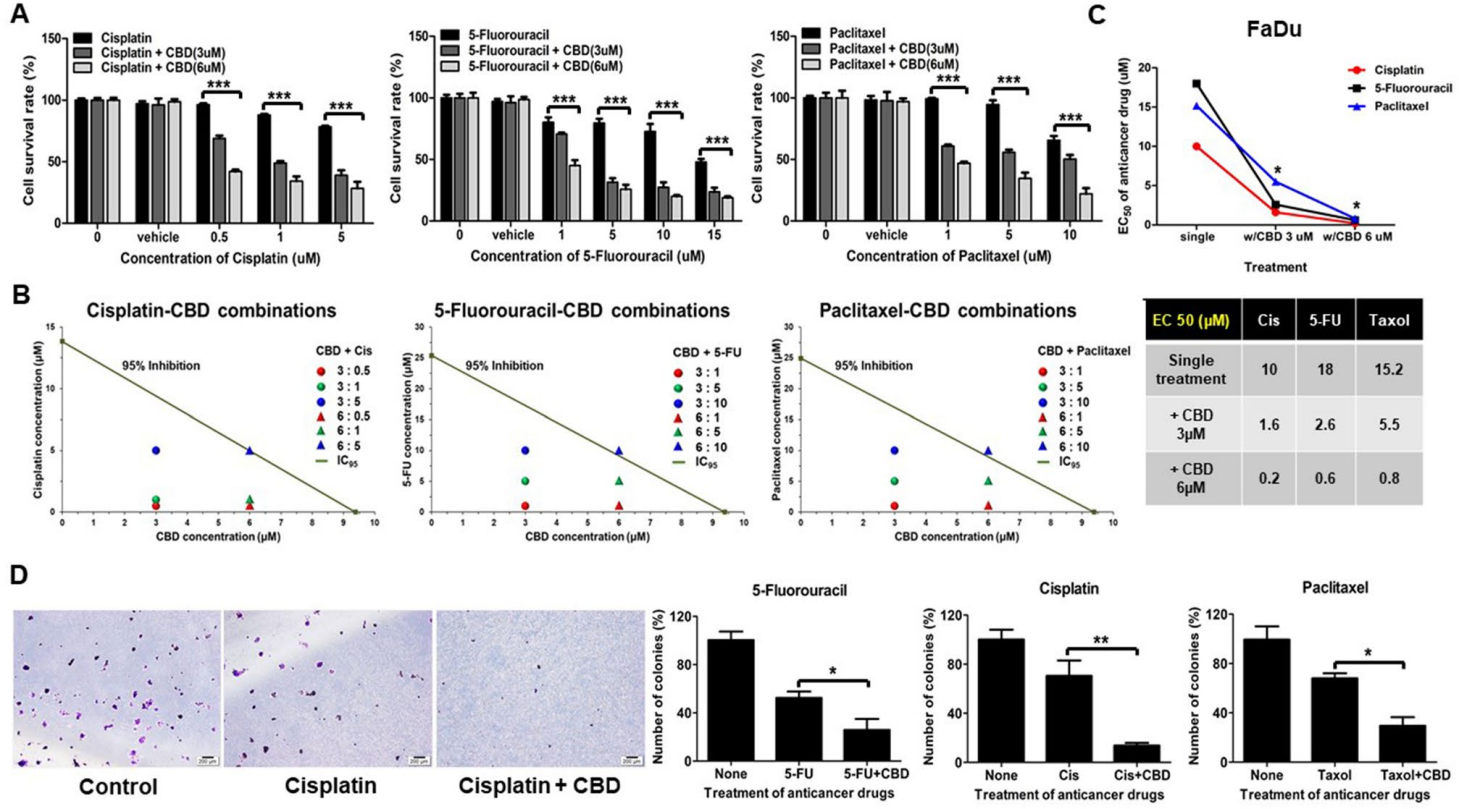
Cannabidiol (CBD) exhibited synergistic efficacy with anti-cancer drugs. (**A**) FaDu cells were treated with various concentrations of Cisplatin, 5-Fluorouracil, and Paclitaxel for 24 h and then cell viability was determined by CCK8 assay to determine EC_50_ value of each drug. After four hours of pretreatment with Cisplatin, 5-Fluorouracil, or Paclitaxel with FaDu cells were exposed to 3 or 6 µM of CBD in each drug group. The Cell survival rate was determined using CCK8 assay at 24 h. (**B**) Isobologram method was used to analyze the additivity and/or synergy of in vitro drug combination treatment in FaDu cells. The diagonal line delineated 95% growth inhibition. Combination Index (CI) values determined using the 50% of inhibitory concentration (IC_50_) values from each drug combination, which were pointed by colored rounds or triangles. Antagonism (CI > 1) and synergy (CI < 1) were indicated above and below the line, respectively. (**C**) Single treatment of Cisplatin, 5-Fluorouracil, or Paclitaxel were compared with combination treatment with 3 or 6 µM of CBD and represented EC_50_ value as the graph and table. (**D**) FaDu cells were exposed to EC_50_ value of Cisplatin (10 µM), 5-Fluorouracil (18 µM), and Paclitaxel (15 µM) alone or combination with 6 µM of CBD and then cultured for 2 weeks. The cells were stained with crystal violet and visualized by a light microscopy. Scale bars, 200 μm. Error bars indicated mean and SD; *p < 0.05; **p < 0.01; and ***p < 0.001 compared with control.

General anti-cancer chemotherapy drugs including Cis, 5-Fluorouracil (5-FU), and Paclitaxel (Taxol) decreased the survival rate of FaDu cells in a dose-dependent manner (Supplementary Fig. S1); specifically, 10 µM Cis, 18 µM 5-FU, or 15.2 µM Taxol in single treatments were determined to be the effective concentrations for 50% death in HNSCC cell lines (Fig. 3B lower panel). To assess whether CBD enhances the efficacy of anti-cancer drugs, we compared the cell survival rate for single treatment with a general anti-cancer chemical drug with survival rates upon combinational treatment with CBD (Fig. 3A). The lower panels of Fig. 3B showed strong synergism in FaDu cells when drug combinations were below 6:5 or 6:10 ratio of CBD:Cisplatin or CBD:5-Fu and/or Paclitaxel, respectively. Combination with 3 µM or 6 µM of CBD markedly inhibited the viability of FaDu cells compared with single therapy of anti-cancer drugs, showing that a significantly lower amount of Cis, 5-FU, and Taxol was sufficient to kill 50% of HNSCC cells (Fig. 3C). To analyze the long-term effect of CBD combination with anti-cancer drugs on clonogenic survival of treated FaDu cells, colony forming assays were performed. Compared with single treatment with 5-FU, Cis, or Taxol, co-treatment with CBD was more effective for anti-cancer therapy (Fig. 3D and Supplementary Fig. S2). Taken together, these results indicated that cytotoxic drug combinations with CBD were more effective in cancer treatments.

### CBD induces apoptosis and autophagy in HNSCC cells

**Figure 4:**
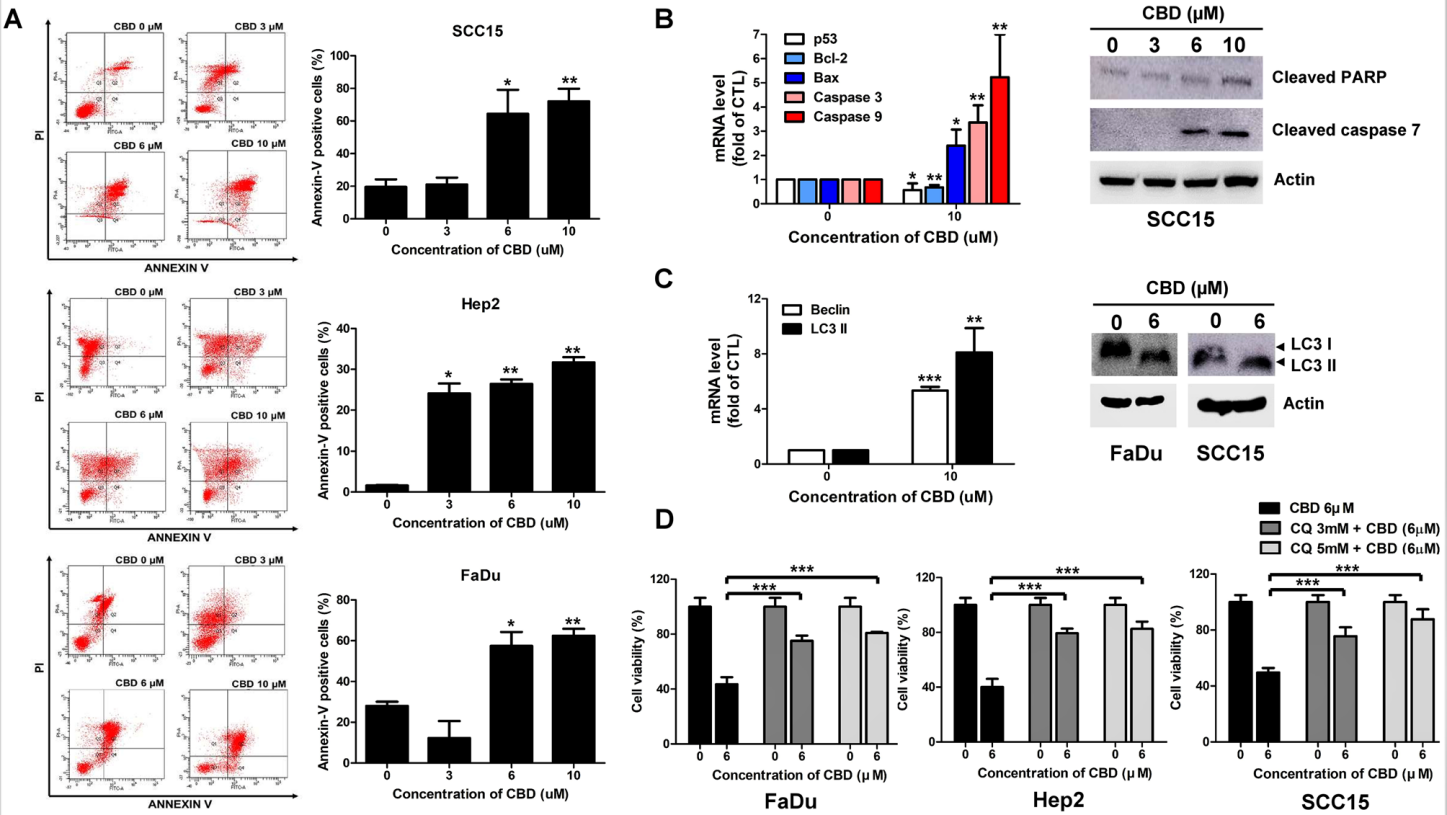
Cannabidiol (CBD) induced human head and neck squamous cell carcinoma (HNSCC) cell death via apoptosis and autophagy. (**A**) SCC15, Hep2, and FaDu cells were treated with 0, 3, 6, or 10 µM of CBD for 24 h and stained with Annexin V and PI. Apoptosis was detected by flow cytometry. (**B**) SCC15 cells were incubated with CBD at the indicated doses and then quantitative real time RT-PCR and western blot analysis were performed. The mRNA levels of p53, Bcl-2, Bax, caspase-3, and -9 and the protein levels of cleaved PARP and caspase-7 were analyzed upon exposure to CBD in HNSCC cells. (**C**) The expression levels of Beclin- and LC3II-encoding genes were determined by quantitative real time RT-PCR after treatment with 10 µM CBD for 24 h. Protein levels of LC3II were determined in FaDu and SCC15 cells treated with 0 or 6 µM CBD for 24 h. (**D**) Pretreatment of FaDu, Hep2, and SCC15 cells with CQ were exposed to 0 or 6 µM CBD for 24 h. The cell viability was determined by CCK8 assay. Data were represented as mean and standard deviation (SD) (n = 3); *p < 0.05 compared with the corresponding control. Full-length gels and blots in (**B**) and (**C**) are included in a supplemental experimental procedure.

We found that CBD significantly inhibited proliferation of HNSCC cells in this study. However, the cellular responses induced by CBD in HNSCC cells, especially cell death signaling, still remained unknown. To examine whether CBD triggered apoptotic signaling in HNSCCs, we first performed Annexin V/PI staining of the three HNSCC cell lines untreated or treated with CBD. CBD dose-dependently remarkably increased the number of Annexin V/PI double positive stained cells compared with numbers in untreated HNSCC cells, indicating that CBD stimulated apoptosis in HNSCC cells (Fig. 4A). Another experiment, shown in Fig. 4B, revealed that treating SCC15 cells with 10 µM CBD decreased the mRNA levels of *TP53* and *Bcl-2,* while levels of *Bax* and *caspase-3* and *-9* were significantly elevated (Fig. 4B, left panel). Among these apoptotic markers, CBD treatment decreased p53 expression, meaning that low levels of p53 expression may be involved in cell cycle arrest, but not apoptosis, when HNSCC cells are exposed to CBD^18^. Immunoblot analysis also revealed that CBD dose-dependently increased the expression of cleaved-PARP and -caspase 7 in SCC15 cells (Fig. 4B, right panel). Next, we investigated whether autophagy signaling-mediated cell death was involved in CBD-induced cytotoxicity of HNSCCs. Increased expression levels of autophagy marker genes, including Beclin*-* and LC3II-coding genes were observed in CBD-treated HNSCC cells (Fig. 4C, left panel). We also clearly detected an increase in LC3II protein level in CBD-treated FaDu and SCC15 cells (Fig. 4C, right panel). However, CBD-induced autophagy may exert a protective function against HNSCCs. Therefore, we inhibited autophagy by adding chloroquine (CQ) before treatment with CBD to HNSCCs and then performed CCK8 assays to determine cell viability. Treatment with CQ resulted in a significant increase in the viability of CBD-treated FaDu, Hep2, and SCC15 cells (Fig. 4D), implying that CBD-induced autophagy enhanced cell death in HNSC cells, but was not a protective mechanism. Overall, CBD-mediated toxicity toward HNSCC cells involves both apoptosis and autophagy mechanisms in cell death signaling.

### Genome-wide changes triggered by CBD in HNSCCs: DNA damage and cell cycle arrest

**Figure 5:**
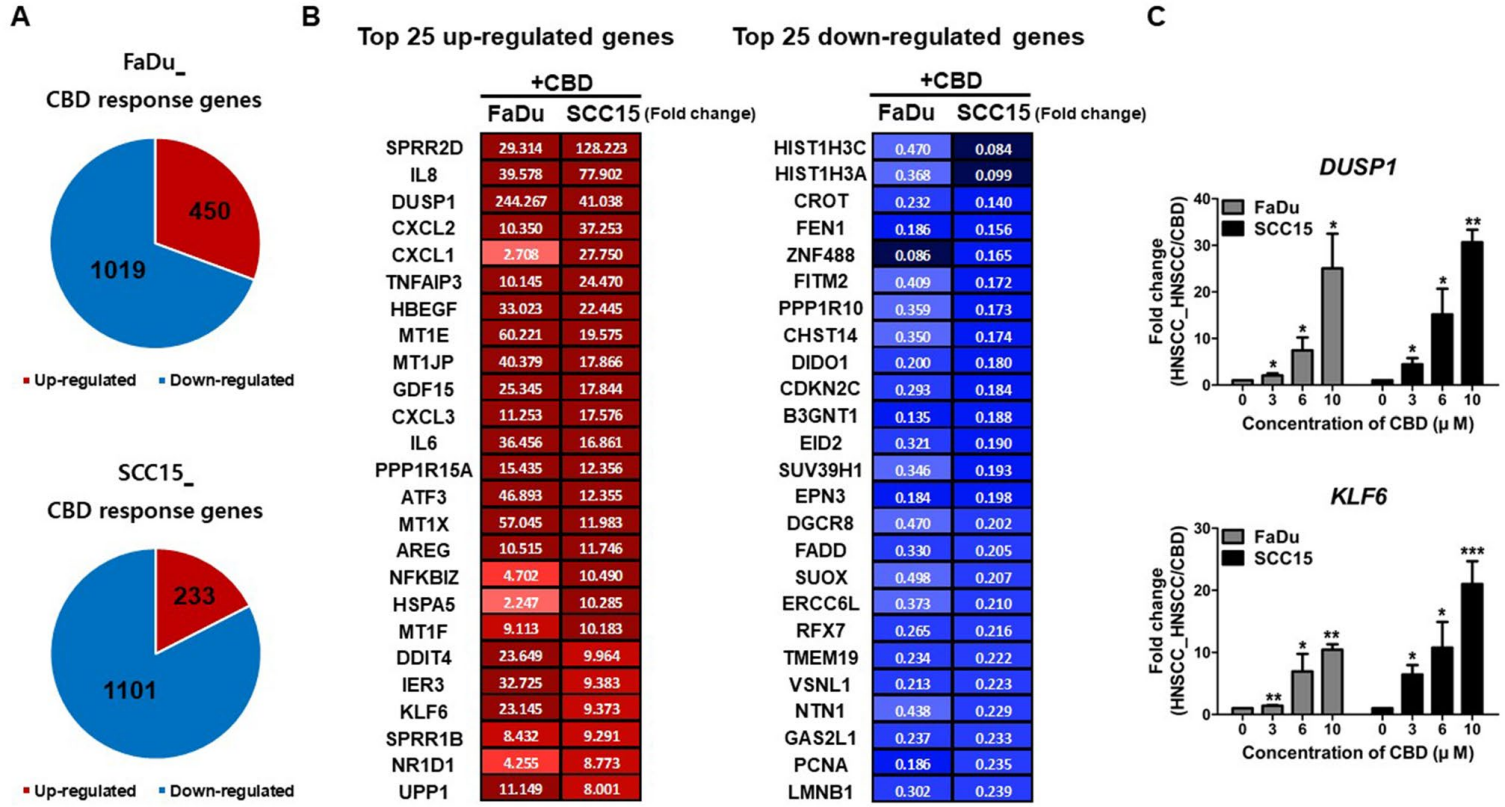
Cannabidiol (CBD) triggered widespread transcriptome changes in head and neck squamous cell carcinoma (HNSCC) cells. (**A**) FaDu and SCC15 cells were treated with 10 µM CBD and RNA-seq was performed to investigate differentially expressed genes (DEG) by CBD treatment on HNSCCs. The number of up- and down-regulated genes in response to CBD in FaDu and SCC15 cells is indicated, respectively. (**B**) The Top 50 up- and down-regulated genes in both FaDu and SCC15 were represented by color images and value of fold change (twofold difference with *p* < 0.05). Color depicted the levels of fold change. (**C**) The representative up-regulated genes encoding DUSP1 and KLF6 in CBD-treated FaDu and SCC15 cells were confirmed by quantitative real-time RT-PCR. Data indicated mean and SD; *p < 0.05; **p < 0.01; and ***p < 0.001 compared with the corresponding control.

**Figure 6:**
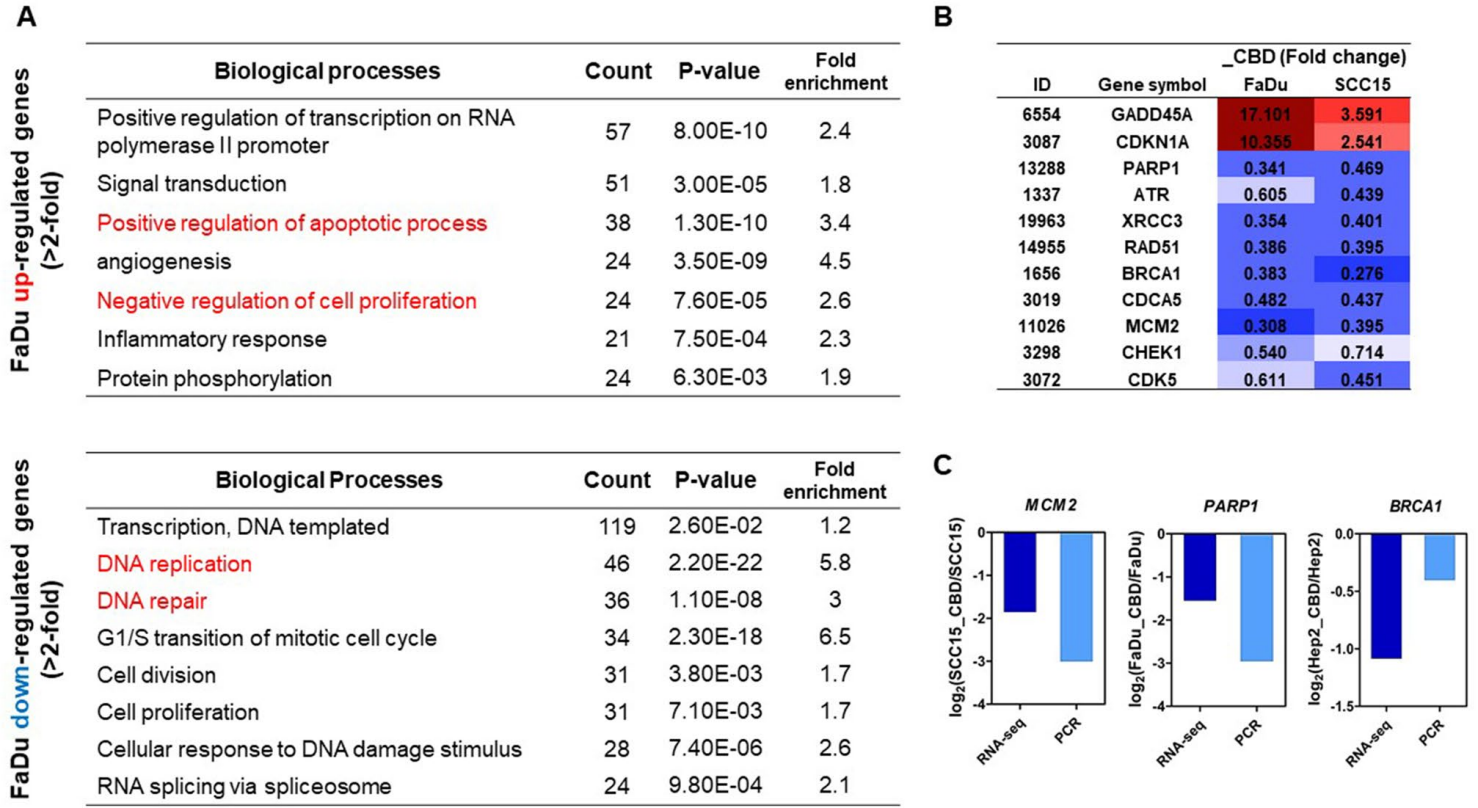
Alteration of Biological processes by cannabidiol (CBD) treatment. (**A**) The significant gene ontology terms of associated biological processes from differentially up-and down-regulated genes in CBD-treated FaDu cells were listed (twofold difference with *p* < 0.05). (**B**) The list showed the DNA damage- and cell cycle arrest-related genes in RNA-seq data. (**C**) The expression levels of down-regulated genes encoding MCM2, PARP1, and BRCA1 were confirmed by quantitative real-time RT-PCR and compared with the results of RNA-seq.

We next investigated the changes at the whole-transcriptome level triggered by CBD treatment in HNSCCs. RNA-seq analysis identified 1469 and 1334 genes in FaDu and SCC15 cells, respectively, as significantly differentially expressed genes (DEGs; < twofold) in CBD-treated HNSC cells. For FaDu cells, 450 genes were significantly up-regulated, while 1019 genes were down-regulated. CBD-treated SCC15 cells exhibited 233 significantly upregulated genes and 1101 down-regulated genes (Fig. 5A). Panel B in Fig. 5 shows the combined top 25 up- and down-regulated genes in FaDu and SCC15 cells. From the up-regulated genes in both cell lines, we selected two genes, *DUSP1* (Dual specificity phosphatase 1) and *KLF6* (Kruppel like factor 6), as downstream targets of EGFR signaling and then confirmed the dose-dependent increase in expression levels of both genes in CBD-treated HNSCCs (Fig. 5C). Additionally, we analyzed the top 50 up- and down-regulated genes in FaDu and SCC15 cells, respectively (Supplementary Fig. S3) 60 cancer-related genes in CBD treated FaDu and SCC15 cells found in our analysis (SI 1). For the DEGs up- and down-regulated by twofold or greater in CBD-treated FaDu cells, the key gene ontology (GO) terms in biological processes included positive regulation of apoptosis, negative regulation of cell proliferation, and down-regulation of DNA replication and repair (Fig. 6A). GADD45A (growth arrest and DNA damage-inducible protein alpha) is involved in DNA repair and apoptosis processes^19^. CDKN1A (cyclin-dependent kinase inhibitor 1A), also known as p21, is associated with DNA damage-linked cell cycle arrest^20^. Importantly, strong up-regulation of *GADD45A* and *CDKN1A* gene expression was observed in both CBD-treated FaDu and SCC15 cells (Fig. 6B), suggesting that CBD might induce apoptosis and cell cycle arrest by modulating GADD45A and p21 levels in HNSCC cells. In addition, cell cycle and DNA damage/repair related genes such as *PARP1* (poly ADP-ribose polymerase 1), *ATR* (ATM and Rad3-related), *XRCC3* (X-ray repair cross-complementing group 3), *CDK5* (cyclin dependent kinase 5), and *CHEK1* (checkpoint kinase 1) exhibited significantly reduced expression levels in CBD-treated HNSCC (Fig. 6B). Down-regulated expression levels of *MCM2* (mini-chromosome maintenance complex component 2), *PARP1*, and *BRCA1* (breast cancer type 1) genes in CBD-treated HNSCC were confirmed by quantitative real-time RT-PCR and then compared with the RNA-seq results (Fig. 6C). Other significantly expressed genes related to DNA repair, cell cycle, cell proliferation, and cell migration in CBD-treated HNSCC were also identified by our data (SI 2).

## Discussion

HNSCC is etiologically caused by exposure to carcinogens such as tobacco and alcohol, and infection with the human papillomavirus (HPV). As for the primary treatment, combining cytotoxic drugs and radiotherapy is the only available therapeutic strategy for patients with HNSCC^6^. A small population of cancer cells regrows and expands locally or forms distant metastases according to three pathophysiological steps: invading, transporting, and recurring^21^. Local recurrence and cancer metastasis are current challenges for treating HNSCC^6^. In order to inhibit and effectively kill cancer cells, one of the recently suggested options for anticancer chemotherapy mainly involves anticancer drug cocktails using cytotoxic and cytostatic chemicals, as well as biotherapies (antibodies, vaccines, gene therapy, and immune therapy, etc.)^22^. Here, we found that CBD has anti-cancer potential as a cytotoxic drug for HNSCC, and combining the natural product CBD with chemotherapeutic drugs such as fluoropyrimidines, platinum analogs, or taxanes might inhibit cancer cell growth significantly more than treatment with single drugs. Cytotoxic drugs for chemotherapy can have several side effects for patients with cancer, including fatigue, nausea, hair loss, and diarrhea because of damage to normal cells. However, CBD can exert a palliative effect and is less toxic to normal cells as a natural compound, and we also revealed that CBD induced cytotoxicity in human HNSCC, but not in normal oral keratinocytes. Therefore, combination treatment with CBD can effectively alleviate uncomfortable side effects in patients with cancer by reducing the use of toxic chemotherapeutic drugs during cancer treatment. In the context of the cancer therapeutic potential of CBD for clinical use, a future study should compare CBD’s effectiveness among primary and advanced/recurrent HNSCC.

It has been known that CBD activates apoptosis-mediated cell death in several cancer cell lines such as breast, colorectal, leukemia, and pancreatic tumor cells^23–26^. Consistently, we also observed that CBD mediated dose-dependent apoptosis in HNSCC. Moreover, we found that treatment with CBD in HNSCC increased levels of cleaved PARP1 and LC3 II, meaning that the cytotoxic effect of CBD in HNSCC is associated with autophagy as well as apoptosis. Inhibiting autophagy prevented CBD-induced cell death in HNSCC in our study, suggesting that CBD-induced autophagy is clearly involved in the cell death mechanism, but not the protective role of autophagy for HNSCC. Several researchers previously demonstrated that autophagy signaling is upstream of apoptosis in the mechanism of cell death induced by CBD^27,28^. We did not explore which cell death mechanism controls CBD-induced cell death, but clearly demonstrated that both apoptosis and autophagy processes are involved in the mechanism of CBD-mediated cell death in HNSCC.

p53 is often inactivated by mutations in most HNSCC cases, and is therefore not able to facilitate the initiation of apoptosis. In our study, the *p53* gene was downregulated by CBD treatment as determined by RNA-seq and PCR. These results might represent a non-functional role of p53 for CBD-mediated apoptosis in HNSCC. However, cleaved PARP1 was observed in CBD dose-dependent manner in HNSCC, which can induce intracellular apoptosis.

Erener et al.^29^ suggested an apoptosis-independent role of caspase 7 and PARP1 cleavage, introducing new insight into inflammatory signaling. The increase in cleaved caspase 7 and PARP1 observed in the present study can be evidence of apoptosis, but it also can facilitate chromatin opening of NF-kB signaling related genes, thereby promoting the expression of proinflammatory genes. Indeed, inflammatory response genes encoding IL-8, IL-6R, NFKBIZ, IL-1R, and PTGS2 were significantly upregulated in our RNA-seq data (SI 3) suggested that CBD-induced cleavage of caspase 7 and PARP1 might stimulate cellular inflammatory responses in HNSCC.

DUSP1 can dephosphorylate both tyrosine and threonine residues as a dual-specificity kinase phosphatase, which is a known negative regulator of EGFR-initiated MAPK (mitogen activated protein kinase) signaling. DUSP1 is capable of inactivating MAPK isoforms in oncogenic Ras/ERK signaling through the activity of dual specific dephosphorylation^30^. Activation of the Ras-mediated MAPK pathway promotes the cell cycle and proliferation, which leads to malignant tumors^31^. Recent studies considered DUSP1 as an anticancer target molecule to modulate the apoptosis and autophagy signaling of over-activated MAPK pathway in cancer cells^32,33^. We also found that *DUSP1* was one of the most upregulated gene by RNA-seq upon treatment of CBD on HNSCC, and confirmed its increased expression levels in this study. The exact mechanism of CBD in HNSCC remains unknown, but our results might suggest that CBD effectively suppresses HNSCC cell proliferation and growth by the inducing the apoptotic and autophagy activity of DUSP1.

Moreover, increased expression levels of *KLF6* were also investigated in our study. KLF6 overexpression has been known to induce apoptosis and inhibit cell proliferation and migration in various cancer cells; one study showed the tumor suppressive function of KLF6 in oral cancer cells^34^. In addition, KLF6 is associated with inactivation of the EGFR-initiated PI3K-mediated signaling pathway by inhibiting AKT and mTOR activation. Actually, EGFR is extensively overexpressed in HNSCC and is considered as an important therapeutic target, but several anti-EGFR-based therapies have not been effective^35^. One of the main issues of EGFR-based therapies in cancer cells is constitutive activation of EGFR-mediated downstream oncogenic signaling (MAPK, PI3K/AKT, JAK/STAT, and AKT/NF-kB pathway) by EGFR mutation, which thereby leads to the development of treatment resistance^36^. Therefore, CBD has great potential as an anticancer drug for HNSCC, not only due to its cytotoxic effect by inducing genotoxic stress, but also because it specifically targeted EGFR terminal mediators by regulating DUSP1 and KLF6 expression. Our results from RNA-seq with the in vitro and in vivo findings suggested that CBD might induce the expression of DUSP1 and KLF6, following the HNSCC tumor suppression via the DUSP1 and/or KLF6 dependent activation of apoptosis and autophagy cellular signaling on HNSCC.

**Figure 7:**
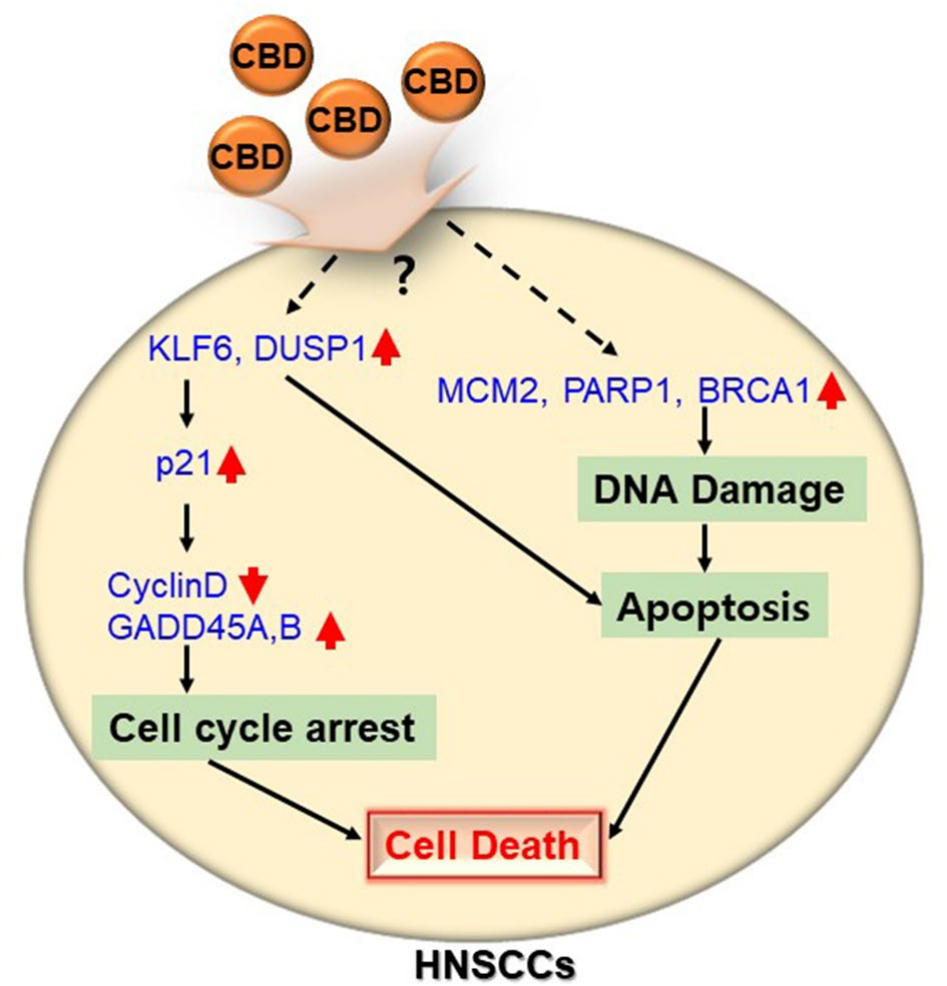
Schematic representation of the proposed anti-cancer mechanisms by CBD treatment on HMEECs.

Interestingly, CBD-treated FaDu cells exhibited significantly downregulated DNA replication and repair processes, confirming dysregulated expression levels of related genes such as *MCM2*, *PARP1*, and *BRCA1*. BRCA1 and PARP1 play a critical role in repairing double- and single-stranded DNA breaks, respectively^37^. In addition, MCM2 is a replicative helicase that initiates proper DNA replication^38^. If DNA damage and replication become uncontrolled because of a lack of key factors involved in DNA repair and replication processes, DNA damage increases, subsequently causing apoptosis in cancer cells^5^. It is still unclear how CBD interferes with DNA replication and repair processes, but our study suggested a possible mechanism of action of CBD on HNSCC: CBD-induced DNA damage via the upregulation of MCM2, PARP1, or BRCA1 can be attributed to induce apoptosis and CBD also subsequent activation of KLF6, which might promote CDKN1A (p21) expression in cells. Increased p21 protein expression consequently triggers cell cycle arrest via the inhibition of cyclin D and stimulation of GADD45A/B expression (Fig. 7).

In conclusion, our study determined the anti-tumorigenic potential of CBD. In addition, single treatment of CBD or co-treatment with chemotherapeutic agents promoted HNSCC cell death along with apoptosis and autophagy processes. Therefore, our study suggests that CBD can be an excellent therapeutic agent against HNSCC.

## Materials and methods

### Cell line and culture procedure

Human oral keratinocytes (HOK) were used as a control cell line against to HNSCC as described^4^, which obtained from ScienCell (#2610) and cultured with oral keratinocyte growth medium supplemented with penicillin/streptomycin (1000 U/mL, Gibco, New York City, NY, USA). HNSCC cells including FaDu (KCLB-30043), Hep2 (KCLB-10023), and SNU-899 (KCLB-00899) were purchased from Korean Cell Line Bank (KCLB, www.cellbank.snu.ac.kr) and SCC15 were obtained from the American Type Culture Collection (ATCC, Manassas, VA, USA; CRL-1623). FaDu, Hep2, and SCC15 cells were cultured in low-glucose DMEM (Lonza, Walkersville, MD, USA) with 10% fetal bovine serum (FBS) (Gibco), and 1000 U/mL penicillin/streptomycin (Gibco). SNU-899 cell lines were maintained with RPMI-1640 medium (Lonza) supplemented with 10% FBS and penicillin/streptomycin (1000 U/mL, Gibco). All cells were sub-cultured at approximately 90% confluence and maintained at 37 °C with 5% CO_2_.

### Reagents and cell viability assay

CBD (Sigma Aldrich, St. Louis, MO, USA; C7515) was provided by Prof. Dr. Sang Cheul Oh from the Korea University of Guro hospital, Korea. CBD powder was dissolved in absolute ethanol (EtOH) and then stored at − 20 °C until use. For cell viability assays, HNSCC and HOK cells were seeded (1 × 10^4^ cells/well) in 96-well plates, and then cultured in growth medium for 24 h. Cells were exposed to CBD in serum-free medium for 24, 48, or 72 h under the indicated conditions and then cell viability was measured using the Cell Counting Kit 8 (CCK-8) (Dojindo Laboratories, Kumamoto, Japan), according to the manufacturer’s protocol, as described^39^. Anti-cancer drugs including Cisplatin (C2210000), 5-Fluorouracil (5-FU) (F6627), and Paclitaxel (Taxol) (T1912) were purchased from Sigma and dissolved in 0.9% sodium chloride, EtOH, and DMSO, respectively. Chloroquine (CQ) was also obtained from Sigma Aldrich (C6628), solubilized in EtOH, and stored at room temperature until use.

Isobologram analysis were performed as previously described^40^. The Combination Index (CI) values were calculated based on the interaction of chosen two drugs at the half of inhibitory concentration (IC_50_) and then indicated antagonism (CI = 1), additivity (CI > 1), or synergy (CI < 1) on graphically presented Isobologram analysis. ED50 plus v 1.0 software was used to determine the of the IC_50_ each drug as previously described^41^.

### Cell migration and invasion assays

For cell migration and invasion assays, 6.5-mm transwells with 8.0-µm pore polycarbonate membrane inserts (BD Biosciences, Franklin Lakes, New Jersey, USA; 3403) were used in this study, following the protocol as described^42^. Briefly, cells were pretreated with CBD for 24 h as indicated at different concentrations and then 1.5 × 10^4^ cells/well in 100 µL of serum-free DMEM media (Lonza) were added in the upper chamber. For invasion assays, 10 µg/mL of Matrigel (Gibco) was added into the upper chamber and polymerized at 37 °C for 2 h, and then the indicated amounts of cells were seeded. The lower chamber of transwells were filled with 600 µL of media containing 20% FBS. Cells were allowed to migrate or invade for 24 h, and then migrated cells were stained with Diff-Quick (Sysmex, Kobe, Japan; 24606) stain, following the protocol as described^43^, and observed under a microscope.

### Trypan blue staining assay

FaDu cells (2 × 10^4^ cells/well) were seeded in 24-well plates, and maintained in complete media for 24 h. The next day, cells were treated with 0, 0.25, 2.5, 10, or 20 µM CBD for 24 h. Prepared trypan blue solution (0.4%) (Sigma; T8154) was used to distinguish between viable and non-viable cells.

### Mouse xenograft tumor model

Animal studies were approved by the Institutional Animal Care and Use Committee (IACUC) and all animals were maintained in a Specific Pathogen-Free (SPF) animal facility according to the guidelines of the IACUC of Korea. Six-week-old female Immunodeficient mice (BALB/c) were purchased from Orient Bio (Seoung-Nam, Republic of Korea) and utilized in this in vivo study. Five mice were tested in each group. FaDu (2 × 10^6^) cells were subcutaneously injected or into the tongue and mice were then housed for 3 days. For the subcutaneously implanted tumor model, CBD was administered orally at 5 mg/kg body weight for 4 times in a week. Mice implanted with FaDu cells into their tongue were added to CBD (5 mg/kg) by intraperitoneal (i.p) administration 3 times per week. Cisplatin (2.5 mg/kg) was intraperitoneally injected every week. The tumor size was determined every 2–3 days and calculated using the formula: V = L × W^2^/2 as described^44^. Tumors were weighed after animals were sacrificed at 4 weeks.

### Colony formation assay

FaDu (1 × 10^2^) cells were seeded in 6-well plates, and then treated with CBD or anti-cancer drugs. After 2 weeks, colonies were fixed in 4% paraformaldehyde for 5 min and stained with crystal violet (Sigma; V5265). The colonies were calculated and normalized to the number of colonies in the untreated control group.

### Apoptosis assay

Apoptosis assay for HNSCCs was performed using a FITC Annexin V/Propidium Iodide (PI) detection kit (BD Biosciences) as described^42^, and determined by flow cytometry. After treatment with 0, 3, 6, or 10 µM CBD for 24 h, the cells were harvested and mixed with Annexin V and PI in the dark for 30 min at room temperature.

### RNA extraction and quantitative real time RT-PCR

RNA was obtained from HNSCC using TRIZOL reagent (Invitrogen, Carlsbad, CA, USA) to analyze differentially expressed gene. cDNA reverse-transcribed using a PrimeScrip 1st strand cDNA synthesis kit (Takara Bio, Tokyo, Japan) from 1 μg RNA, according to the manufacturer’s instructions. Quantitative real-time polymerase chain reaction (qRT-PCR) was carried out in an ABI Prism 7300 Detection System (Applied Biosystems) using ABI SYBR green dye (Applied Biosystems, Foster City, CA, USA). The 2^(−∆∆Ct)^ method was used to analyze relative expression levels of mRNA and values were normalized to that of *GAPDH*^45^. The specific primer sequences are shown in a supplemental experimental procedure.

### Western blot analysis

HNSCC cells were cultured in 6-well culture plates with 5 × 10^5^ cells/well and exposed to CBD in FBS-free medium and further incubated for 24 h. Collected cells were lysed and then same amount of proteins were loaded by 8–12% sodium dodecyl sulfate polyacrylamide gel electrophoresis. The proteins were transferred to membranes, which were then blocked with 5% (w/v) skim milk contained TBST (494 mM Tris, 2.74 M NaCl, 54 mM KCl, adjusted to pH 7.4 using HCl) for 30 min at room temperature. The membrane blots were probed with primary antibodies against cleaved PARP (1:1000; Cell Signaling Technology, Danvers, Massachusetts, USA; 5625S), cleaved Caspase-7 (1:1000; Cell Signaling Technology; 8438S), LC3 (1:1000; Cell Signaling Technology; 4108S), and β-actin (1:2000; Santa Cruz Biotechnology, Dallas, Texas, USA; sc-47778). The following day, the membranes were incubated with a secondary antibody against horseradish peroxidase-conjugated anti-rabbit or anti-mouse IgG (Invitrogen, 1:5000) for 1 h at room temperature and then images were captured using a Fusion Solo Imaging System (Vilber Lourmat, Marne-La-Vallée, France). Full-length gels and blots are included in a supplemental experimental procedure.

### RNA-seq

Total RNA was isolated from FaDu, Hep2, and SCC15 cell lines either untreated or treated with CBD using TRIZOL reagent (Invitrogen) and then quantified in a Nanodrop ND-2000 spectrophotometer. RNA purity was checked using Agilent 2100 Bioanalyzer. Ion Torrent sequencing libraries were prepared, according to the AmpliSeq Library prep kit protocol (Thermo Fisher, Part# MAN0010742) as described^46^. An Ion AmpliSeqTM Transcriptome library was constructed with the Ion Transcriptome Human Gene Expression Kit (Thermo Fisher, SKU # A26325) as per manufacturer’s protocol. 50 ng of total RNA was used for cDNA library preparation and amplified by the Ion AmpliSeq Transcriptome Human Gene Expression kit (over 20,000 human RefSeq genes). Adapters and barcodes were ligated to the library amplicons followed by magnetic bead purification. The concentrated library was measured using an Ion Library TaqMan Quantitation Kit (Thermo Fisher, SKU#4468802), according to the manufacturer’s procedure. Multiple libraries were combined together with equal molar ratios for one Ion 550 chip and clonally amplified using the Ion Chef System (Thermo Fisher), and then sequenced on an Ion Torrent S5xI machine (Thermo Fisher).

Sequence in Ampliseq transcriptome target regions were read with the Torrent Mapping Alignment Program (TMAP) and sequences were quality-controlled by Casava v1.8.2 Pipeline and Cutadapt v1.6^47,48^. The result genes were normalized, and deferential expression tested using DESeq2 and Bioconductor package^49,50^. The Benjamini and Hochberg correction was used to obtain adjusted *p* values < 0.05. Genes that were up- and down-regulated by twofold or more each were assessed for functional and pathway enrichment analysis, such as Gene Ontology (GO) using the Database for Annotation, Visualization and Integrated Discovery (DAVID) bioinformatics resources^51^. Heatmaps were generated with Multi Experiment Viewer 4.9.0 (MeV 4.9.0)^52^.

### Statistical analysis

All representative data were presented as mean ± standard deviation (SD) from at least in triplicate experiments. Statistical analysis was carried out using the student’s two-tailed t test and one-way ANOVA in SPSS software. *p* values of < 0.05 (*), 0.01 (**), and 0.001 (***) were considered statistically significant.

## Supplementary information

Supplementary Table S1.

Supplementary Table S2.

Supplementary Table S3.

Supplementary Figures.
