# Supplementary material for: Cannabidiol enhances cytotoxicity of anti-cancer drugs in human head and neck squamous cell carcinoma
Source: Sci Rep. 2020 Nov 26;10:20622. doi: 10.1038/s41598-020-77674-y (PMC7692486; doi:10.1038/s41598-020-77674-y)
Supplement: Supplementary file 4 — Supplementary Figures. [file 41598_2020_77674_MOESM4_ESM.pdf]

Cannabidiol Enhances Cytotoxicity of Anti-cancer Drugs in Human Head and Neck Squamous Cell Carcinoma

Yoon Young Go, PhD[1,2], Su Ra Kim[1], Do Yeon Kim[1], Sung-Won Chae, MD[1], Jae-Jun Song, MD[1,2]*

Supplementary figures and figure legends

Figure. S1. Anti-cancer effects of Cisplatin, 5-Fluorouracil, and Paclitaxel on FaDu cells.

FaDu cells were treated with various concentrations of Cisplatin, 5-Fluorouracil, and Paclitaxel for 24 hours and then cell viability was determined by CCK8 assay. Error bars indicated mean and SD; $*p < 0.05$, $**p < 0.01$, and $***p < 0.001$ compared with control.

Figure. S2. Cannabidiol (CBD) exhibited synergistic anticancer effect with 5-Fluorouracil and Paclitaxel.

FaDu cells were exposed to EC_{50} value of 5-Fluorouracil (18 µM) and Paclitaxel (15 µM) alone or combination with 6 µM of CBD and then cultured for 2 weeks. The cells were stained with crystal violet and visualized by a light microscopy. Scale bars, 200 µm.

Figure. S3. Differentially up- and down-regulated genes in HNSCC cell line dependent

The top 50 up- and down-regulated genes in FaDu and SCC15 were represented by images of heatmap clustering (2-fold difference with $p < 0.05$). Color bar depicted the levels of color contrast for heatmap data.

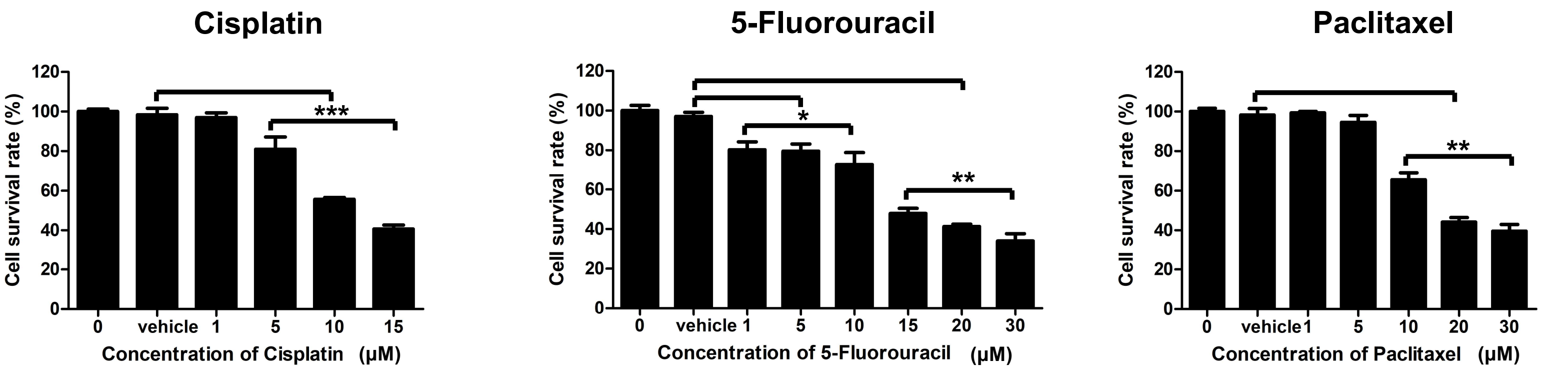

Cisplatin
Cell survival rate (%)
120
100
80
60
40
20
0
***
0
vehicle
1
5
10
15
Concentration of Cisplatin (μM)
5-Fluorouracil
Cell survival rate (%)
*
**
0
vehicle
1
5
10
15
20
30
Concentration of 5-Fluorouracil (μM)
Paclitaxel
Cell survival rate (%)
**
0
vehicle
1
5
10
20
30
Concentration of Paclitaxel (μM)

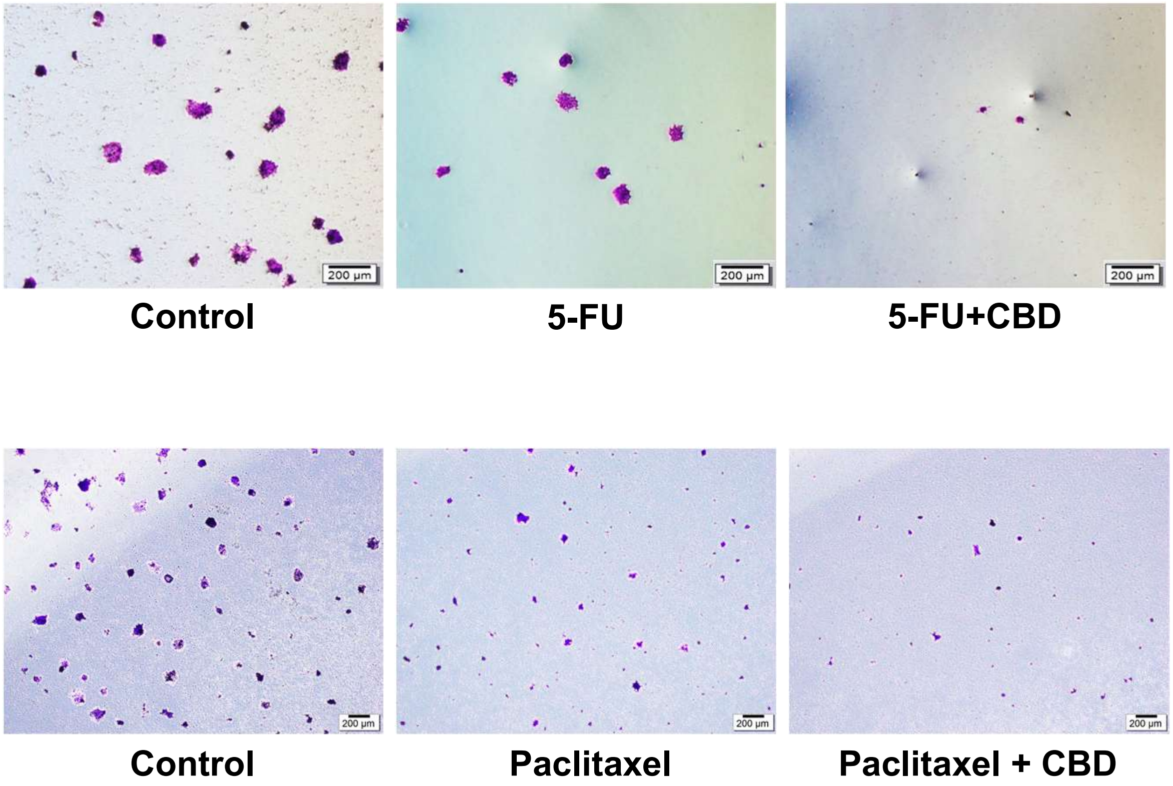
200 µm
Control
5-FU
5-FU+CBD
Control
Paclitaxel
Paclitaxel + CBD

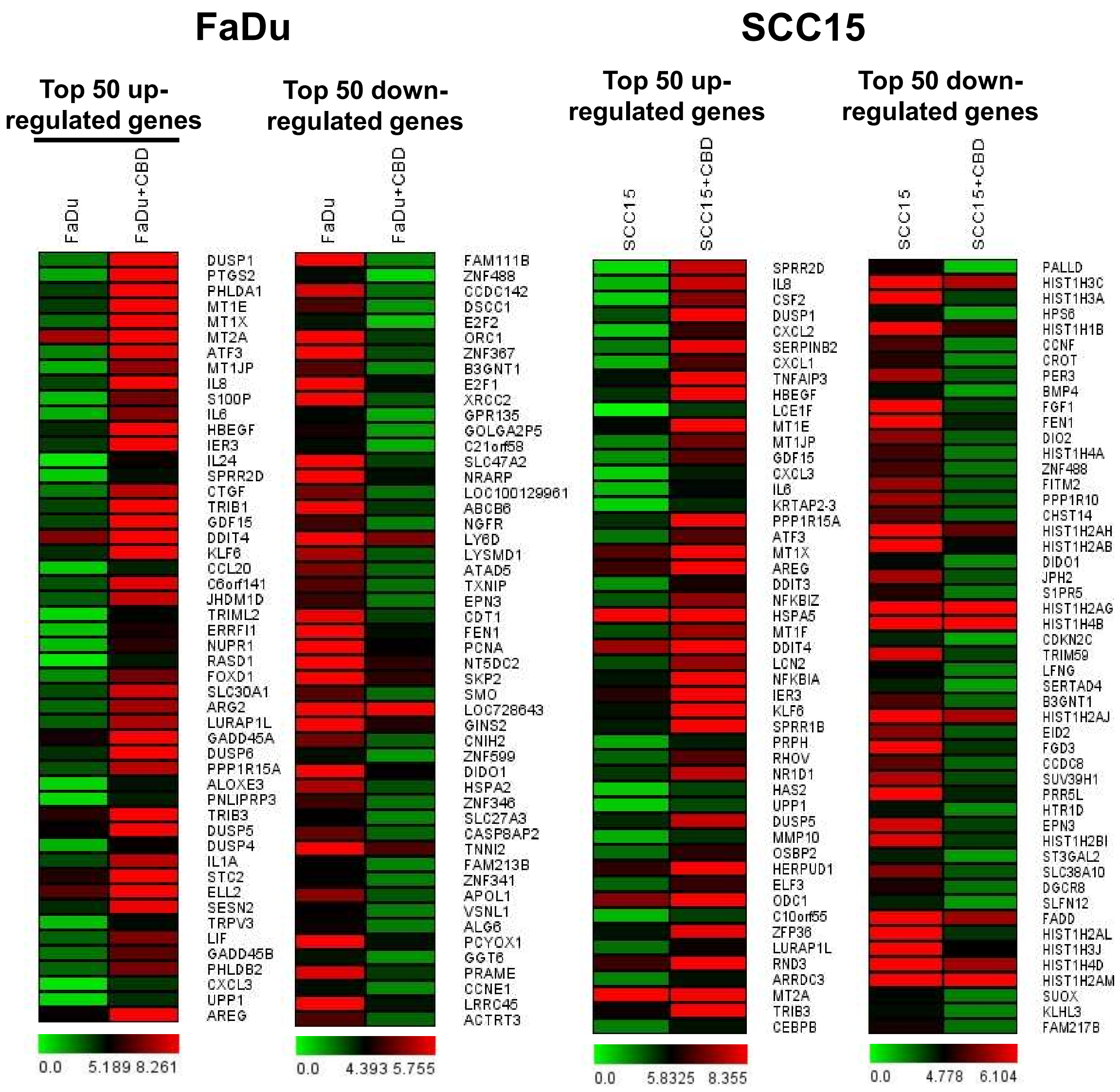
FaDu
SCC15
Top 50 up-regulated genes
Top 50 down-regulated genes
Top 50 up-regulated genes
Top 50 down-regulated genes
FaDu
FaDu+CBD
DUSP1 PTGS2 PHLDA1 MT1E MT1X MT2A ATF3 MT1JP IL8 S100P IL6 HBEGF IER3 IL24 SPRR2D CTGF TRIB1 GDF15 DDIT4 KLF6 CCL20 C6orf141 JHDM1D TRIML2 ERRFI1 NUPR1 RASD1 FOXD1 SLC30A1 ARG2 LURAP1L GADD45A DUSP6 PPP1R15A ALOXE3 PNLIPRP3 TRIB3 DUSP5 DUSP4 IL1A STC2 ELL2 SESN2 TRPV3 LIF GADD45B PHLDB2 CXCL3 UPP1 AREG
0.0 5.189 8.261
FaDu
FaDu+CBD
FAM111B ZNF488 CCDC142 DSCC1 E2F2 ORC1 ZNF367 B3GNT1 E2F1 XRCC2 GPR135 GOLGA2P5 C21orf58 SLC47A2 NRARP LOC100129961 ABCB6 NGFR LY6D LYSMD1 ATAD5 TXNIP EPN3 CDT1 FEN1 PCNA NT5DC2 SKP2 SMO LOC728643 GINS2 CNIH2 ZNF599 DIDO1 HSPA2 ZNF346 SLC27A3 CASP8AP2 TNNI2 FAM213B ZNF341 APOL1 VSNL1 ALG6 PCYOX1 GGT6 PRAME CCNE1 LRRC45 ACTRT3
0.0 4.393 5.755
SCC15
SCC15+CBD
SPRR2D IL8 CSF2 DUSP1 CXCL2 SERPINB2 CXCL1 TNFAIP3 HBEGF LCE1F MT1E MT1JP GDF15 CXCL3 IL6 KRTAP2-3 PPP1R15A ATF3 MT1X AREG DDIT3 NFKBIZ HSPA5 MT1F DDIT4 LCN2 NFKBIA IER3 KLF6 SPRR1B PRPH RHOV NR1D1 HAS2 UPP1 DUSP5 MMP10 OSBP2 HERPUD1 ELF3 ODC1 C10orf55 ZFP36 LURAP1L RND3 ARRDC3 MT2A TRIB3 CEBPB
0.0 5.8325 8.355
SCC15
SCC15+CBD
PALLD HIST1H3C HIST1H3A HPS6 HIST1H1B CCNF CROT PER3 BMP4 FGF1 FEN1 DIO2 HIST1H4A ZNF488 FITM2 PPP1R10 CHST14 HIST1H2AH HIST1H2AB DIDO1 JPH2 S1PR5 HIST1H2AG HIST1H4B CDKN2C TRIM59 LFNG SERTAD4 B3GNT1 HIST1H2AJ EID2 FGD3 CCDC8 SUV39H1 PRR5L HTR1D EPN3 HIST1H2BI ST3GAL2 SLC38A10 DGCR8 SLFN12 FADD HIST1H2AL HIST1H3J HIST1H4D HIST1H2AM SUOX KLHL3 FAM217B
0.0 4.778 6.104

Supplemental Experimental Procedures

Table 1. Sequences of oligonucleotide primers for quantitative RT-PCR.

Primer	Direction	Sequence
p53	forward	5'-AGACCTGTGGGAAGCGAAAA-3'
	reverse	5'-TCATCCATTGCTTGGGACGG-3'
Bcl-2	forward	5'-TCCCTCGCTGCACAAATACTC-3'
	reverse	5'-ACGACCCGATGGCCATAGA-3'
Bax	forward	5'-CCAAGGTGCCGGAACTGA-3'
	reverse	5'-CCCGGAGGAAGTCCAATGT-3'
Caspase3	forward	5'-ATTGTGAGGCGGTTGTAGAA-3'
	reverse	5'-CATGTATGATCTTTGGTTCCAA-3'
Caspase9	forward	5’-TGCTGAGCAGCGAGCTGTT-3’
	reverse	5’-AGCCTGCCCGCTGGAT-3’
Beclin	forward	5’-AGCTGCCGTTATACTGTTCT-3’
	reverse	5’-TGTGTCTTCAATCTTGCCTT-3’
LC3	forward	5’-GCAGCCTTTCTTCCAGAGAC-3’
	reverse	5’-CTGGAAAAGTGGAGGCTGAG-3’
DUSP1	forward	5’- CGTCTCAGCCAATTGTCCCA-3’
	reverse	5’- AACACCCTTCCTCCAGCATTC-3’
KLF6	forward	5’-ACGGCTGCAGGAAAGTTTACA-3’
	reverse	5’-GCTTTCGGAAGTGCCTGGTTA-3’
MCM2	forward	5’-ATATCAGCGCAACCGCTTTG-3’
	reverse	5’-AGGTCGTGGCTGAACTTGTT -3’
PARP1	forward	5’-GGTGGACATCGTTAAAGGAACCAA -3’

		5’-TCCTCCTTGGACGGCATCT-3’
BRCA1	forward	5’-CTGAAAGCCAGGGAGTTGGT-3’
	reverse	5’-CTTGTTTCACTCTCACACCCAGAT-3’
GAPDH	forward	5'-TCGCCCCACTTGATTTTGG-3'
	reverse	5'-GCAAATTCCATGGCACCGT-3'

Full-length blots in Figure 4B

Anti-cleaved PARP

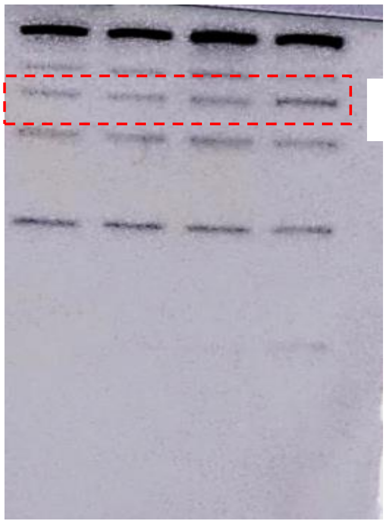

Crop here

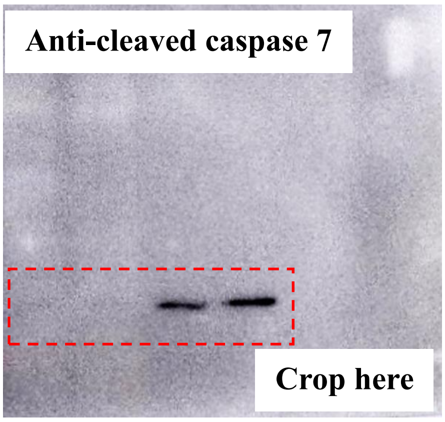

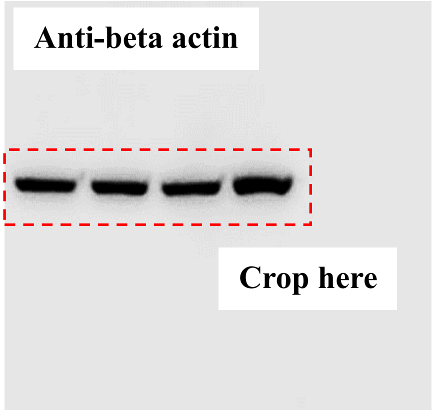

Original blots

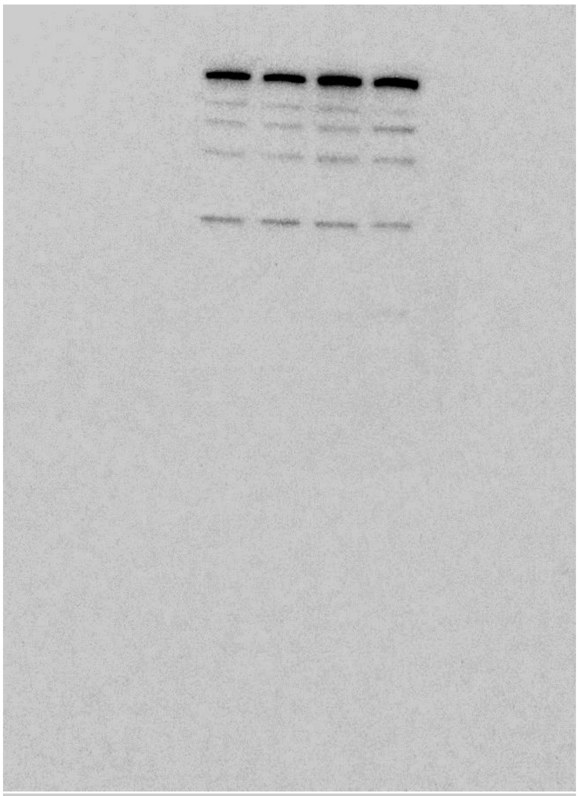

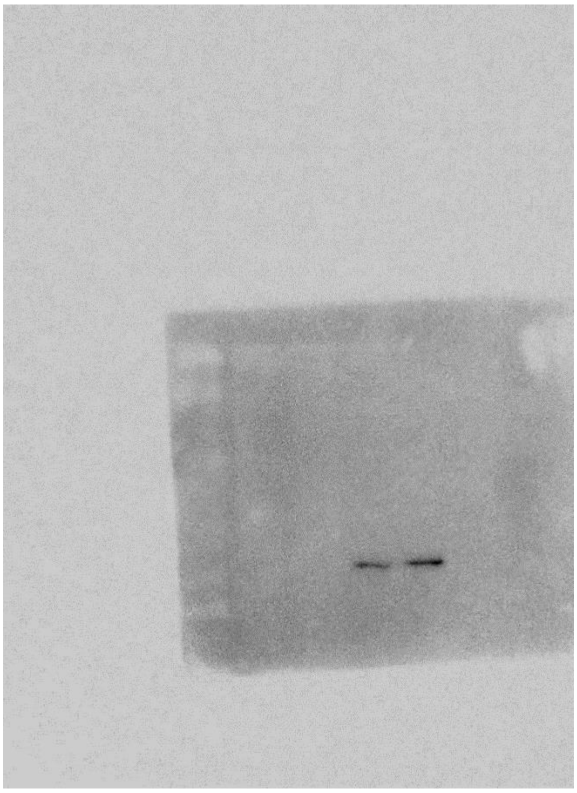

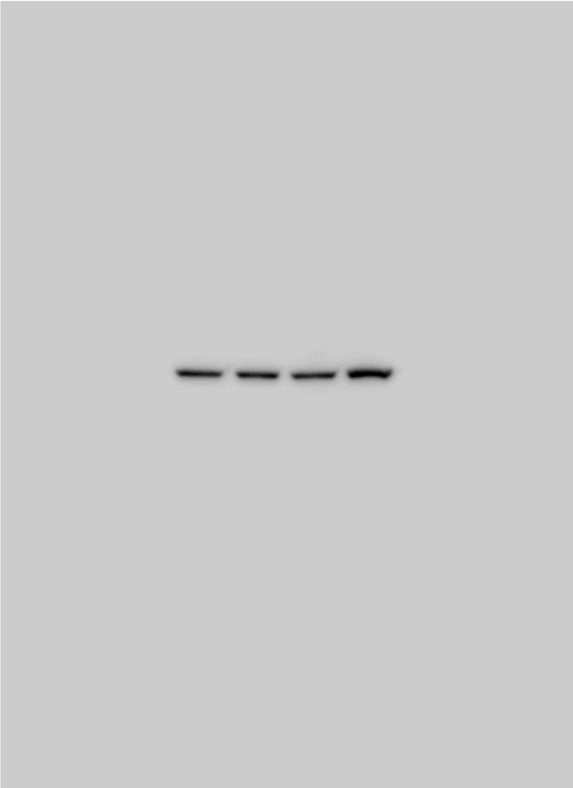

Full-length blots in Figure 4C

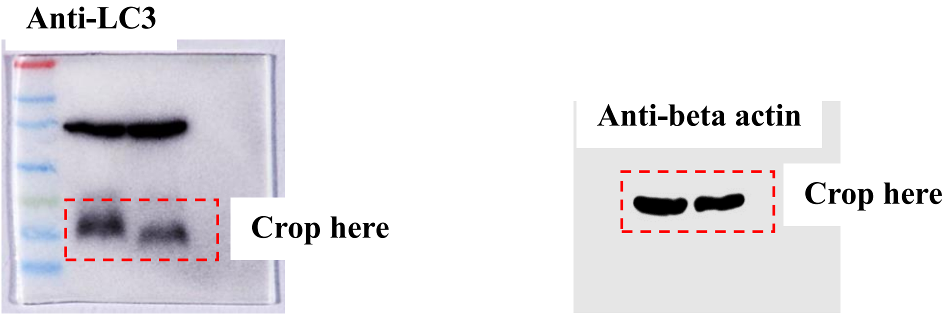

<FaDu>

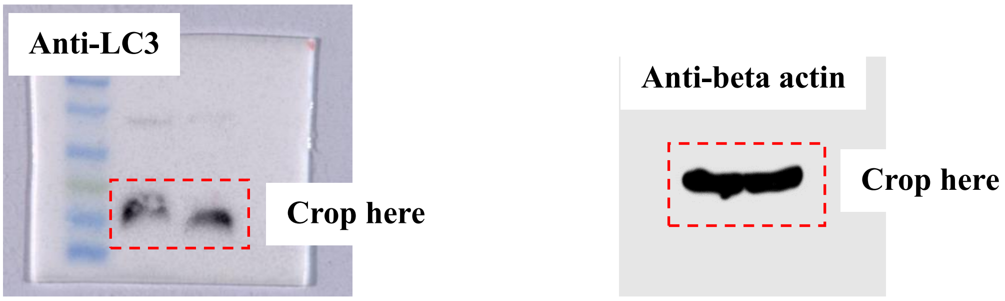

<SCC15>

Original blots with multiple exposure images

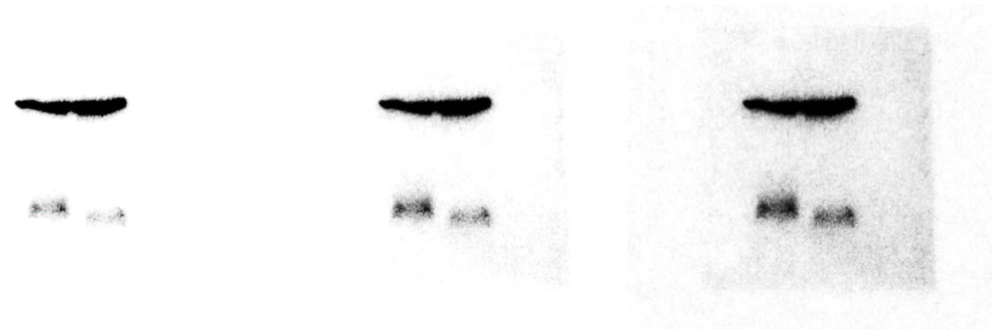

<FaDu anti-LC3>

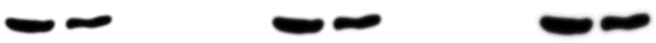

<FaDu anti-actin>

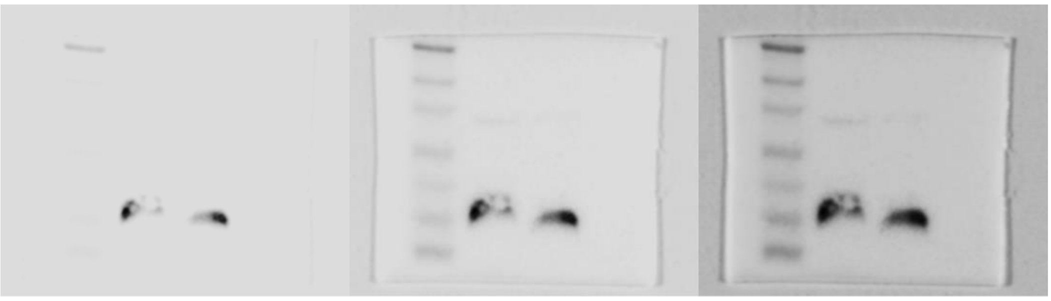

<SCC15 anti-LC3>

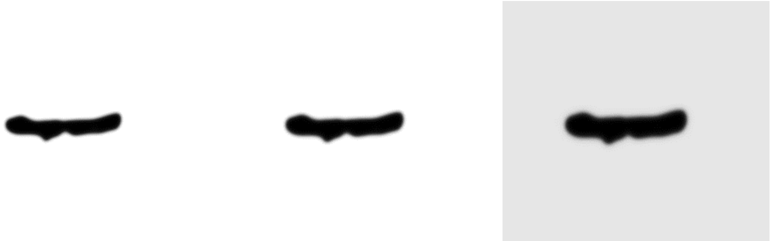

<SCC15 anti-actin>
